# A Narrative Review of the Factors Affecting the Mental Health of Adolescents and Young People During the COVID-19 Pandemic

**DOI:** 10.7759/cureus.66781

**Published:** 2024-08-13

**Authors:** Ketan Tamirisa, Hima B Maringanti

**Affiliations:** 1 Public Health, Washington University in St. Louis, St. Louis, USA; 2 Cognitive Artificial Intelligence, Maharaja Sriram Chandra Bhanja Deo (MSCB) University, Baripada, IND

**Keywords:** anxiety, lockdown, depression, covid-19, coronavirus, youth mental health outcomes, youth mental health, youth psychology

## Abstract

Coronavirus disease 2019 (COVID-19), caused by the severe acute respiratory syndrome coronavirus 2 (SARS-CoV-2) virus, led to a worldwide pandemic. There were unprecedented changes in the mental health of children, adolescents, and youth in the age group of 8-18 years as a result of COVID-19. The objective of this review is to identify direct and indirect factors that influenced these changes. We identified three main groups of factors that could have impacted the mental health of young people during the pandemic: (i) familial factors, (ii) social and educational factors, and (iii) positive variables. Our review suggests that the COVID-19 pandemic negatively impacted the mental health of children and adolescents through stressors like social isolation, parental unemployment and loss, and disrupted routines. As a result, sadness, hopelessness, depression, and generalized anxiety all increased significantly among adolescents during the pandemic, coupled with a simultaneous increase in substance use, suicide attempts, and violence. However, the pandemic also offered some positive variables such as reduced bullying, more family time, and improved relationships for those with adequate socioeconomic resources. The complex factors affecting the mental health of young people during the pandemic underscore the necessity for additional research to comprehend their individual effects. We advocate for collaborative youth-centered initiatives involving educational organizations, mental health experts, policymakers, healthcare systems, and other community stakeholders to effectively tackle these challenges.

## Introduction and background

The coronavirus disease 2019 (COVID-19) pandemic, caused by the severe acute respiratory syndrome coronavirus 2 (SARS-CoV-2) virus, profoundly impacted global health, both physical and mental. Officially declared a pandemic by the World Health Organization (WHO) on March 11, 2020, the crisis led to staggering statistics by December 23, 2022, with over 6.99 million deaths and 772.05 million confirmed cases worldwide [1]. In the United States, the Centers for Disease Control and Prevention (CDC) reported a troubling increase in persistent sadness and hopelessness among high school students, which rose from 26% in 2009 to 36% during the early pandemic period [2]. Similarly, research conducted by Stony Brook University revealed that rates of depression and generalized anxiety disorder among adolescents nearly doubled during the pandemic when compared to pre-pandemic levels [3]. The impact was further reflected in the significant rise of other outcomes, including adolescent drug overdoses, suicide attempts, and violence in 2020 compared to the previous year, with pediatric emergency room visits for mental health crises increasing by 69% [4, 5].

A cross-sectional study conducted in Iraq highlighted that survivors of COVID-19 experienced moderate levels of anxiety, with younger individuals exhibiting notably higher levels of stress (Estimate: 18.96, p < 0.001) and anxiety (Estimate: 2.79, p = 0.001) than older age groups [6]. The neuropsychiatric effects of the virus on the central nervous system may also contribute to mental health issues [7]. However, an interesting observation is that the prevalence of depression was lower among youth who tested positive for COVID-19 compared to those who tested negative (1% vs. 1.7%; 95%CI 0.53-0.80, adjusted p < 0.001) [8]. This suggests that while direct viral effects are a factor, they are not the sole contributors to the mental health crisis among young people.

Youth are particularly vulnerable to mental health challenges, as 75% of mental health disorders manifest by the age of 25 [9]. The pandemic’s societal changes, such as the transition to virtual learning platforms and decreased peer interactions, have had a substantial impact on youth mental health [10]. These changes disrupted normal socialization, routine, and educational experiences, exacerbating feelings of isolation and stress among adolescents. Additionally, socio-cultural barriers and stigma surrounding mental health issues often prevent young people from seeking timely and appropriate care [11]. This underscores the urgent need for more comprehensive data and the development of early, youth-centered therapeutic strategies to address these challenges.

The efficacy of interventions such as cognitive behavioral therapy (CBT) has been well studied and proven, emphasizing the importance of an interdisciplinary approach that includes community education and healthcare system engagement [12]. Other effective solutions require collaborative efforts from educational organizations, mental health professionals, policymakers, and healthcare systems to create a supportive environment for youth. This review seeks to delve into the multifaceted factors that influenced changes in the mental health of young people during the COVID-19 pandemic, aiming to identify gaps and opportunities for improving support mechanisms and resilience for this vulnerable population. By understanding these factors in depth, we can better prepare for future crises and enhance the overall mental health support systems for young people.

## Review

Methodology 

This review is based on an extensive PubMed search of relevant articles. The search strategy focused on identifying studies published between 2020 and 2024 that examined the impact of COVID-19 on the mental health of young people and adolescents. Search terms included "youth mental health," "adolescents," "COVID-19," "psychological impact," and "risk factors." Relevant articles were screened based on their title, abstract, and full text, with a particular emphasis on studies discussing factors influencing mental health outcomes among young people during the pandemic to build this commentary. The terms children, adolescents, youth, and young people used in this review primarily denote the age group of 8-18 years.

Results

Our analysis of the pertinent literature resulted in the identification of key agents that potentially contributed to changes in the mental health of adolescents and young people during the pandemic. We organized these factors into three main groups based on thematic commonality: (i) Family influences, including parental/caregiver death, unemployment, and mental health struggles, (ii) Social and educational factors, including societal measures, school closures and virtual schooling/ issues with inaccessibility, and (ii) Support network and effects, such as increased family interaction and reduced bullying rates. We identify these factors, describe relevant changes in them as a result of the pandemic, and discuss the extent of their impact on the mental health of young people. 

Family Influences

Parent/caregiver death: Globally, 10.5 million youth lost their parents and caregivers due to COVID-19 up to May 1, 2022 [13]. In the United States, over 140,000 youth under 17 years of age lost one or both parents/caregivers from 2020 to early 2021 [14]. Older children, especially those aged 10 and older, were more likely to be orphaned, partly due to higher mortality among people aged 50 and above [15]. Unwin et al. found that from March 2020 to October 2021, the number of youth who lost caregivers due to COVID-19 rose by five million. The last six months of their study period saw a near doubling of these losses, likely due to more infectious virus strains and reduced protective measures post-vaccine availability [16]. Parental loss varied by region and ethnicity. For instance, Peru had 10 times the rate of parental loss compared to the United States [17]. In the United States, Hispanic, Black, and American Indian/Alaskan Native youth were 2.1-3.55 times more likely to lose caregivers than non-Hispanic White youth. Contributing factors include higher exposure to infection, poor living conditions, limited healthcare access, and systemic racism [18]. Although the pandemic caused unprecedented parental loss, there is no conclusive evidence linking this directly to increased mental health problems in the young population. A longitudinal study in Cape Town, South Africa (OCAY Study), is examining the specific impact of caregiver loss on the mental health of young people during the pandemic [19]. Positive support from remaining caregivers or adults can mitigate the negative effects of parental loss [20]. However, the pandemic has also strained adults' mental health, potentially reducing their ability to support affected youth.

Parent/caregiver unemployment: During the COVID-19 pandemic, economic instability among parents and caregivers significantly impacted the mental health of young people. The overall unemployment rate in the United States was higher than in the Great Recession in 2010, affecting 21% of youth who had at least one jobless parent in April 2020 [21]. By July 2021, 31.3 million Americans reported recent unemployment due to business closures, and many faced prolonged illness post COVID-19, affecting their ability to work and care for children [22,23]. Unemployment or underemployment was not limited to job unavailability. During the COVID-19 pandemic, around 16 million working-age Americans continued to suffer from the prolonged sequelae after COVID-19. Around two to four million adults could not work due to persistent sickness. These parents had difficulty caring for their children as well [19]. This widespread unemployment contributed to economic struggles and increased the risk of poverty. In fact, the pandemic pushed 97 million more people into extreme poverty (living on less than $1.90 per day), with disproportionate effects on low-income and marginalized communities [24,25]. Economic struggles led to increased family conflict, parental stress, and reduced academic performance among youth. Studies showed that parental unemployment was linked to higher rates of psychological and physical abuse, particularly in lower-income families [26,27]. Parental joblessness was associated with increased rates of persistent sadness and suicide attempts among adolescent students [28]. In Colorado, youth with unemployed caregivers experienced higher rates of emotional and physical abuse compared to those with employed caregivers. This evidence suggests that economic instability and job loss during the pandemic were associated with adverse mental health outcomes and increased maltreatment among youth [29].

Parent/caregiver mental health struggles: Parental mental health deteriorated during the COVID-19 pandemic, affecting children. The Harris Poll by the American Psychological Association found that parents or caregivers were twice as likely to report stress compared to adults without children [30]. Parenting stress rose from 3.5% before the pandemic to 22.4% in May 2020 and remained elevated at 12.2% in September 2020 [31]. Early in the pandemic, 40% of parents experienced moderate to severe anxiety and depression due to COVID-19-related stressors such as disrupted routines, increased responsibilities, online schooling demands, financial difficulties, and health concerns [32]. Studies in the United States, Mexico, and South America during the COVID-19 pandemic found associations between parental distress and youth mental health issues [33-35]. Several factors, including parental health and lifestyle, parenting quality, forced changes in daily routines, and severe financial instability have been identified that linked parental mental health to the mental health of young people during the pandemic [31,36]. According to CDC data, emotional abuse by parents or caregivers affected 55% of United States high school students in 2021, up from 13.9% pre-pandemic, and physical abuse rose to 11%, up from 5.5% [37]. From these studies, it is evident that parental mental health issues had negative coupling effects on the mental well-being of youth during COVID-19.

Social and Educational Factors

Societal measures: Societal measures were implemented to reduce social contact and reduce the spread of infection. Schools nationwide implemented measures such as mask mandates and social distancing policies (sitting six feet apart in classrooms). These measures and policies led to increased social isolation and loneliness, impacting the mental health of young people severely. For example, in two different cohort studies, social distancing during the COVID-19 pandemic was shown to increase psychological stress, loneliness, anxiety, fear, and feelings of uncertainty in youth [38,39]. 

School closures and virtual schooling: Educational instruction for many youths changed, with a switch to virtual platforms from traditional in-person classes. Globally, 1.38 billion learners experienced school closures [40]. During the height of the social isolation measures, 80% of United States school-aged youth were in virtual schooling [40]. Even when schools reopened, many implemented additional measures to curb infection rates, like reducing after-school activities. Societal measures to curb COVID-19 spread led to increased social isolation and loneliness, impacting mental health in young people. Curtailed after-school activities and sports had several negative impacts on children and adolescents, including increased non-educational screen time (social media, television, and games), contributing to reduced sleep time, withdrawal from family interactions, and behavioral aggression [41,42]. Virtual schooling was associated with worse mental outcomes compared to in-person or hybrid models [43]. A survey of adolescents (13-19 years) reported higher stress (44.7% vs. 25%) and persistent depression (19.1% vs. 7.6%) in virtual learners [44]. School closures deprived youth of essential support from friends and non-family adults, exacerbating mental health issues. Although a study from Italy reported that virtual school did provide both academic and social benefits, allowing teenagers to stay somewhat connected during this time, the benefits were not uniformly seen [45]. A population survey in Japan investigated the effect of school closures on suicide rates among adolescents in early COVID-19 and no significant change in suicide rates was reported during the school closure months. The main limitation of this study is the inability to establish direct causality between school closures as an independent factor and suicide rates [46, 47]. A longitudinal survey of adolescents in the United Kingdom reported that mental health and overall well-being were influenced by the pre-pandemic connectedness with the school and peers. Reductions in anxious symptoms were noted in those youth who were least connected with their peers at school [48].

Virtual inaccessibility: Disparities in access to virtual learning platforms became more apparent during the pandemic. Black and Hispanic households, and those from lower-income families, faced significant disadvantages due to a lack of internet access and digital literacy (79% White households vs. 66% Black households and 61% Hispanic households) [49]. Additionally, youth from low-income families also had issues with accessing virtual or broadband educational platforms. This inequity worsened school performance and increased stress and isolation among youth [50,51]. Those who needed special education services in school before the pandemic were also affected negatively by school closures due to the loss of therapy and expert guidance from special education teachers [52]. In addition, inaccessibility limited the opportunity to connect with friends virtually, likely increasing social isolation.

Positive Variables and Their Effects

Increased family support: The COVID-19 pandemic was a complex stressor for youth and parents. However, there were some factors that positively impacted the mental health of the youth. For example, some families reported lower rates of discord and reduced child abuse and domestic violence due to increased “at-home” time [53,54]. An Australian survey found that 77% of youth experienced both positive and negative impacts, citing increased personal space, social connection, self-reflection, and wellness time as supportive reasons [55]. This parental “at-home” time led to increased bonding between youth and their parents/caregivers, leading to improvements in mental health. A cross-sectional survey of over 4,000 children and adolescents aged 6-17 years in China reported some positive impacts during COVID-19: reduced school time and teacher monitoring, and increased time for personal stuff and parental-bonding time. These factors led to 21.4% of participants experiencing an increase in life satisfaction compared to pre-pandemic time. In the study, home quarantine was perceived as more positive than negative [56].

Reduced bullying rates: In-school bullying rates dropped by 40%, and some studies noted reductions in cyberbullying, though others did not [57, 58]. For instance, a cross-sectional survey of Asian youth conducted in 2016, 2019, and 2021 reported increased cyberbullying rates (16.7%, 17.2%, and 23.2%, respectively), potentially due to pandemic-related targeting [59]. Conversely, a University of Ottawa study found bullying rates dropped from 60% to 40% and cyberbullying from 13.8% to 11.5% [60]. Another study using Google trends combined with in-school instructional modes found that bullying rates dropped by 27% and cyberbullying rates dropped by 20% among adolescents in the United States [61]. Similar results of a reduction in cyberbullying were also seen in Korea between 2019 and 2020 [62]. Additionally, youth who experienced cyberbullying during the pandemic reported being able to cope better due to increased parental support at home [48,61,63]. Overall, cyberbullying rates were mostly better during the pandemic in Western countries but seemed to increase in Asian countries [48].

Discussion

Positive Variables

While the pandemic led to a deterioration of youth mental health overall, there were some positive variables that resulted. There are many factors that also could have influenced mental health in adolescents and youth during the pandemic including financial stability, parental adaptation to remote work, and digital literacy. Changes in educational and work structures during the pandemic might have enabled open parent-child discussions, which were crucial in buffering fear and anxiety. The pandemic presumably exacerbated socioeconomic divides, as affluent families likely saw more protective factors due to less stress, financial security, increased access to virtual technology, and more parental support. Overall, youth experienced mixed mental health impacts during the pandemic, shaped by multiple factors. More research is needed to fully understand these dynamics.

Potential Remedies and Future Implications

Based on current insights from the pandemic, preparing for future crises and safeguarding the mental health of children and adolescents requires a comprehensive, multifaceted approach. We recommend several proactive measures for the future to mitigate adverse impacts on their mental health.

Implementing comprehensive community interventions: Interventions like CBT have proven successful in improving mental health and addressing harmful thoughts when operating within the Stepped Care Model framework, which is tailored to the specific needs of individuals and encompasses four levels of care: basic, intermediate, advanced, and critical [12]. To achieve similar success on a community-wide scale, comprehensive interventions that involve multiple community stakeholders must be implemented. This includes collaboration between healthcare providers, educators, local organizations, and policymakers to create a cohesive support network to ensure that mental health resources are accessible and effective.

Enhancing socioeconomic support: Initiatives should be implemented in low-income zip codes that provide financial, social, and health-based resources to parents. This can be achieved through targeted psychoeducational interventions focused on improving parents’ emotional resilience, coping skills, and relationships with their children. Furthermore, interventions that enhance open and supportive communication between family members should be promoted. Enhancing support structures can help reduce mental health problems for both parents and children, strengthening family cohesion. Ultimately, a supportive family structure can serve as an effective buffer against adverse life events and future crises.

Improving educational support: Academic institutions should incorporate mental health-based courses into their regular curricula. Students should become equipped with essential tools and strategies to confront mental health issues in a healthy way. Additionally, teachers, faculty, and staff should be trained on trauma-informed practices, help expand access to on-campus mental health services, and establish on-campus support groups. Establishing a conducive and inclusive learning environment would help address the broader socioemotional needs of students beyond purely academic support. 

Utilizing digital health: Technology should be leveraged to connect youth and adolescents with mental health resources. Teletherapy-based programs, online counseling platforms, and digital self-help tools should be implemented at school-based health centers, outpatient clinics, and hospitals in underserved zip codes in order to expand access to mental health resources for youth. Implementing digital health interventions in low-income areas will help improve mental health outcomes among youth.

Advocating for policy change: Advocating for policy change to prioritize mental health in youth and adolescents is important. This can be achieved through advancing pertinent legislative reforms on a local, state, or national level. Additionally, change can occur through advocating for funding to increase mental health access, resources, and interventions for young people. Strengthening policy frameworks will help promote timely, equitable access to mental health care. 

Further research is needed to evaluate the effectiveness of these interventions. It’s unlikely that employing a siloed approach will mitigate most pandemic-related mental health issues in youth. Because mental health is driven by multiple factors, we need society-wide approaches from different angles, as well as individualized support for youth and their families. As evidenced by current data, some youth fared better with better adaptation to the pandemic than others. This is most likely due to the interplay between numerous familial, societal, and social determinants of health factors that affect mental health. Although the long-term effects of COVID-19 on the mental health of the young remain unclear, targeted strategies and individualized interventions are needed to enhance preparedness for future crises.

Study Limitations

While our review provided a comprehensive, thoughtful cover of changes in the mental health of young people and adolescents and potential contributing factors as a result of the COVID-19 pandemic, there exist some limitations. Since our review is not systematic, we have not identified and screened all existing literature. Thus, we acknowledge a selection bias. Furthermore, most of the studies in this review were cross-sectional and failed to include longitudinal data points. While these one-time surveys added to the description of the mental health impact during COVID-19, they cannot establish causal relationships. Additionally, our review includes the United States and global data and does not consider the regional differences and interplay of many region-specific factors that may have influenced the mental health of the youth or adolescents.

Much is still to be uncovered about the mental health fallout of the COVID-19 pandemic. For example, we do not know which or a combination of stressors were the main drivers of adverse mental health in youth. While we have some evidence that certain groups, such as those who live in poverty, are from underrepresented racial and ethnic groups, or face academic and social disadvantages, were affected disproportionately, it is yet to be determined why these groups suffered more. Although we commented on the fact that parental loss was one of the factors that worsened mental health in children and adolescents, we acknowledge that it is a logical corollary and is not based on any direct studies during the pandemic. However, our article remains a significant contribution to existing literature, providing an in-depth overview of plausible factors that affected changes in the mental health of the young during the COVID-19 pandemic. 

## Conclusions

The COVID-19 pandemic introduced significant stressors for youth, including social isolation, parental or caregiver illness and death, job losses, school closures, reduced social interactions, and declining mental health. Conversely, measures like school closures provided some respite from bullying and improved family relationships for some youth. These benefits were more evident in families with better virtual access and resources. Parental influence is crucial for the mental health of children and adolescents. Effective communication and support from parents during the pandemic were essential. Supporting parents economically, physically, and mentally can indirectly protect youth.

The long-term effects of the pandemic on the mental health of young people remain a concern. Disrupted milestones and the return to pre-pandemic routines require ongoing support. The complexity of factors influencing youth mental health during the pandemic highlights the need for further research to understand their independent impacts. Understanding these key factors is crucial for guiding societal actions in future crises, resource allocation, and ensuring families/schools are better prepared. Collaborative youth-centered efforts among educational organizations, mental health experts, policymakers, healthcare systems, and other community stakeholders are essential to address these challenges effectively.
